# The Etiology of Community-Acquired Pneumonia Correlates with Serum Inflammatory Markers in Children

**DOI:** 10.3390/jcm11195506

**Published:** 2022-09-20

**Authors:** August Wrotek, Julita Robakiewicz, Katarzyna Pawlik, Patryk Rudzinski, Izabela Pilarska, Aleksandra Jaroń, Aleksandra Imiełowska, Małgorzata Jarzębowska, Katarzyna Zabłocka, Teresa Jackowska

**Affiliations:** 1 Department of Pediatrics, Centre of Postgraduate Medical Education, Marymoncka 99/103, 01-813 Warsaw, Poland; 2 Department of Pediatrics, The Bielanski Hospital, Cegłowska 80, 01-809 Warsaw, Poland; 3 Student Research Group at the Department of Pediatrics, The Bielanski Hospital, Cegłowska 80, 01-809 Warsaw, Poland

**Keywords:** community-acquired pneumonia, procalcitonin, CRP, RSV, influenza, *Mycoplasma pneumoniae*, *Streptococcus pneumoniae*

## Abstract

Community-acquired pneumonia (CAP) severely affects pediatric hospitalizations. This study assessed the contribution of CAP to hospitalizations, its etiology in relationship with age, and the inflammatory markers. Between 2013 and 2018, 1064 CAP patients were hospitalized and diagnosed with bacterial/possibly bacterial pneumonia (BP), viral/possibly viral pneumonia (VP) and atypical pneumonia (AP). The etiology was confirmed using blood/pleural fluid culture/polymerase chain reaction (PCR), rapid antigen test/PCR in nasopharyngeal swabs, or serological studies. CAP accounted for 9.9% of hospitalizations and 14.8% of patient days. BP was diagnosed in 825 (77.5%), VP in 190 (17.9%), and AP in 49 (4.6%) cases; the confirmed etiology (***n*** = 209; 20%) included mostly influenza (39%; ***n*** = 82), respiratory syncytial virus (RSV, 35%; ***n*** = 72), and *Mycoplasma pneumoniae* (19%; ***n*** = 39). VP frequency decreased with age (41% in < 3 mo to 9% in ≥ 60 mo), in contrast to AP (13% in ≥ 60 mo). Among the analyzed parameters, the best differentiating potential was shown by: C-reactive protein (CRP, AUC_BP-VP_ = 0.675; 95% CI: 0.634–0.715), procalcitonin (AUC_BP-AP_ = 0.73; 95% CI: 0.67–0.794), and CRP/procalcitonin (AUC_AP-VP_ = 0.752; 95% CI: 0.67–0.83); a good positive predictive value (88.8%, 98.3%, and 91.6%, respectively) but a low negative predictive value (29.5%, 13.1%, and 40.7%, respectively) was observed. CAP influences hospital patient days more than the crude number of patients would suggest. On a clinical basis, BP is mainly recognized, although viral pneumonia is confirmed most often. RSV and influenza are responsible for a huge percentage of hospitalized cases, as well as *M. pneumoniae* in children aged ≥ 5 years. Serum inflammatory markers may help differentiate etiological factors.

## 1. Introduction

Lower respiratory tract infections are the major global cause of hospital morbidity and mortality in children, and each year, they are responsible for approximately 12 million hospitalizations and 4.5 million deaths [1,2,3]. According to the World Health Organization, pneumonia (in the vast majority, community-acquired pneumonia (CAP)) causes up to one million fatal cases in children under 5 years of age, which comprises around 15 percent of the total number of deaths in this age group [4,5], although a significant decrease in pneumonia-related mortality has been achieved (from 1.7 million in 2000 to 0.9 million in 2015) [6].

The global estimates report approximately 155 million cases of pneumonia each year, with considerable geographical and socioeconomic variations [7], and the number of hospitalizations is an important issue regarding the use of healthcare resources, since 7 to 13% of children with pneumonia require hospital treatment [8]. In Poland, although pneumonia-related mortality in children is low, CAP also contributes significantly to healthcare resource use; in 2020, respiratory tract infection were responsible for over 90 thousand hospitalizations in children [9]. 

Prior to the introduction of conjugate vaccines, typical bacteria, i.e., *Streptococcus pneumoniae* (*S. pneumoniae*) and *Hemophilus influenzae* type B, were the most common causes of pneumonia [8], yet pediatric vaccination programs managed to decrease its frequency, and more cases of viral pneumonia are now diagnosed; a recent development in diagnostic techniques has also played an important role [10]. Viral etiology is most commonly related with respiratory syncytial virus (RSV), influenza, and adenovirus [11,12,13,14,15,16], and strongly relies on virus detection in upper respiratory tract samples; thus, the risk of a false-positive result related to colonization instead of an infection may be encountered [10]. On the other hand, blood cultures pose the risk of a false-negative outcome, since a positive result is seen only in 2.5–15% of samples [17,18,19,20]. Atypical pneumonia occurs in 10–30% of CAP, and *Mycoplasma pneumoniae* (*M. pneumoniae*), *Chlamydophila pneumoniae* (*C. pneumoniae*), and *Legionella pneumophila* (*L. pneumophila*) are the main pathogens [21].

Children with CAP often require antimicrobial treatment, which is inherently associated with side effects and the development of antimicrobial resistance; a targeted therapy should be sought after to minimize these adverse outcomes [22]. Difficulties with the confirmation of the etiology are met in 15.8–67.3% of cases of clinically confirmed pneumonia [11,12,14,15,16], thus making empirical therapy an everyday clinical practice; the extensive comprehension of local epidemiologic conditions is crucial for appropriate therapy implementation. Serum inflammatory markers, including C-reactive protein (CRP) and procalcitonin (PCT), may provide substantial data for antimicrobial guidance, although evidence in this area of pediatrics is rather scarce, and broader studies are needed [23,24,25]. The procalcitonin (PCT) level increases within 3–4 h after contact with the infectious factor and can reach maximal levels as soon as within 6–24 h, while the CRP increases at a slower pace: it rises after 4–6 h, reaching a maximum within 36–50 h [23,26,27,28]. Procalcitonin seems to be the most promising biomarker in assessing the risk of bacterial infection [24], thus helping to decide on antibiotic implementation, and, according to some studies, also on its cessation, decreasing the risk of unnecessary or prolonged antibacterial treatment [29,30,31,32,33,34]. Atypical etiological factors (*Mycoplasma* and *Chlamydophila pneumoniae*) have been suggested to induce lower PCT levels than typical bacteria [35,36], but an important question on the possible use of increased CRP to PCT ratio in the prediction of *Mycoplasmatic pneumonia* has also recently been raised [37,38].

Except for significant fluctuations and shifts in epidemiological trends, local antimicrobial stewardship and infection management guidelines need to be based upon current data; the local Polish data on the contribution of the particular etiological factors in hospitalized CAP cases, however, are scarce. Moreover, antibiotics are overused and often inappropriately prescribed, making CAP a significant public health concern in Poland [39]. Another rationale behind this study was the fact that, although CAP is one of the most frequent problems, investigations on the practical use of inflammatory markers are still lacking. The study focused on the etiology of the hospitalized CAP cases regarding the age, its impact on the length of stay, and the diagnostic performance of routinely used inflammatory markers. Additionally, the contribution of pneumonia to the total number of hospitalizations and the total number of patient days was estimated. 

## 2. Materials and Methods

This was a retrospective observational study. Electronic medical charts of the patients hospitalized in the period between 2013 and 2018 (6 consecutive years) at the pediatric ward of the Bielanski Hospital, Warsaw, were reviewed, and the data were extracted into a previously prepared grid. The search included the final diagnoses of pneumonia according to the International Classification of Diseases, Tenth Revision (ICD-10): J10.0 (influenza with pneumonia, virus identified), J11.0 (influenza with pneumonia, virus not identified), J12 (viral pneumonia), J13 (pneumonia due to *Streptococcus pneumoniae*), J14 (pneumonia due to *Haemophilus pneumoniae*), J15 (bacterial pneumonia), J16 (pneumonia due to other infectious organisms), J17 (pneumonia in diseases classified elsewhere), and J18 (pneumonia, organism unspecified). All the children diagnosed with CAP were eligible for the study. 

In accordance with the Polish recommendations on the treatment of respiratory tract infections, a clinical diagnosis of CAP is made upon the presence of signs and/or symptoms consistent with pneumonia, which include fever, cough, tachypnea, intercostal retractions, a dull percussion note, crackles or a bronchial murmur on auscultation [40]. According to the Polish guidelines, the presence of at least one of these symptoms may be suggestive of pneumonia, while the presence of abnormalities on auscultation in a feverish coughing child is highly suggestive, but in order to decrease the risk of misdiagnosis, children in our department are diagnosed with pneumonia when at least a combination of two clinical signs and typical auscultatory abnormalities is seen or, in the case of a lack of abnormalities in chest auscultation, a confirmation in an imaging study (chest X-ray or chest ultrasound) is needed [40]. A community origin of the disease was identified if the above signs/symptoms were present prior to hospitalization or up to the first 48 h after hospital admission. The inclusion and exclusion criteria are presented in Table 1.

The etiological factor was established if there was: 1. the growth of the pathogen in a sample from a sterile site (blood or pleural fluid) proven by a culture or its presence by a molecular assay (polymerase chain reaction, PCR), and/or 2. the presence of *Bordetella pertussis*, *Bordetella parapertussis*, or *Bordetella holmesii* in a swab from the upper respiratory tract, and/or 3. the presence of viral pathogens commonly associated with pneumonia (influenza and RSV) in the upper respiratory tract swab, tested using rapid antigen test and/or PCR in a nasopharyngeal swab (in the case of discrepancies between the results, the PCR was judged to be conclusive), and/or 4. positive serological tests suggesting a recent/ongoing infection (positive IgM titer for *Mycoplasma pneumoniae*, *Chlamydophila pneumoniae*, or positive IgA titer against *Bordetella pertussis*). According to the final ICD-10 diagnosis and based upon the etiological factors, patients were finally divided into 3 groups: bacterial/potentially bacterial pneumonia (BP), viral/potentially viral pneumonia (VP), and atypical pneumonia (AP). All the diagnoses were made and encoded with the use of ICD-10 at the discretion of the attending pediatricians responsible for the treatment of the patient, and were not influenced by the study. When a single etiological factor was confirmed, the corresponding etiology of pneumonia was established; when more than one etiological factor was found, corresponding etiologies were diagnosed. In the other cases, the diagnosis was made upon the clinical course, use of and response to the treatment; when antibacterial treatment was considered necessary (and implemented) by a physician, potentially bacterial pneumonia was diagnosed; when a patient was successfully cured and discharged without antibiotics, potentially viral pneumonia was the diagnosis, although no viral factor was established. In fact, this subgroup of the study may also contain bacterial pneumonia cases that did not require antibiotic treatment, but this approach, in the opinion of the authors, might be used, since it translates clearly to practice—patients in this group do not require antibacterial treatment and may benefit from refraining from antibiotics. In the case of atypical pneumonia, only confirmed cases were diagnosed as AP. With regard to treatment success, all the cases were verified in terms of readmission due to the possible complication of pneumonia. Additional separate analysis was also performed in the subgroup with a confirmed etiology. 

The primary outcome was the contribution of the particular etiological factors to the hospitalized CAP cases. The share of the specific etiological agents was presented in the following age groups: <3 months of age; 3–11 months old (mo); 12–59 months of age; and ≥5 years old (yo), i.e., ≥60 mo. Secondary end-points compared the diagnostic performance of routinely used inflammatory markers, including white blood cell count (WBC), the absolute neutrophil count (ANC), the C-reactive protein (CRP), procalcitonin, and the CRP to PCT ratio, in the differentiation between the etiological agents. Additionally, this study assessed the contribution of pneumonia to the total number of hospitalizations and the total number of patient days during the analysis period, the length of stay (LOS) in the 3 study groups, and the LOS according to the particular causative agents.

Laboratory procedures:

Blood was taken immediately after admission, and inflammatory markers were assessed and blood culture performed. In the case of serological studies, a blood sample was taken upon admission and/or during the hospitalization. Nasopharyngeal swabs were taken immediately after admission. WBC and ANC were counted using Sysmex XT2000i, and Sysmex XN1000/Sysmex XN550 (Sysmex Corportation, Kobe, Japan) since 10 April 2014. CRP was determined using the Cobas 6000 analyzer (Roche Diagnostics Ltd., Rotkreuz, Switzerland) with the limit of detection of 0.1 mg/L. Procalcitonin was determined using the Cobas e411 analyzer (Roche Diagnostics Ltd., Rotkreuz, Switzerland) until 4 August 2016, and using the Cobas 6000 analyzer (Roche Diagnostics Ltd., Rotkreuz, Switzerland) from 5 August 2016; the limit of detection was 0.02 ng/mL. Serological studies were performed with the use of enzyme-linked immunosorbent assay (ELISA)—Euroimmun Medizinische Labordiagnostika AG (Luebeck, Germany)—in the case of *Chlamydophila pneumoniae* and with the use of chemiluminescent immunoassays (CLIA)—Liaison XL (DiaSorin S.p.A., Saluggia, Italy)—in the case of *Bordetella pertussis*. The RSV/influenza PCR was performed with the use of RSV Xpert Xpress Flu/RSV XC GeneXpert (Cepheid, Sunnyvale, CA, USA). All the procedures were performed in accordance with the manufacturers’ instructions. 

Data distribution was estimated using the Kolmogorov–Smirnov test. The normally distributed data are presented as the mean ± standard deviation (SD), while skewed data are presented as the median and interquartile range (IQR), and adequate parametric or non-parametric tests were performed (the Student’s *t*-test or the Mann–Whitney U test, respectively). One-way ANOVA or a non-parametric Kruskal–Wallis test with corresponding post hoc tests (Bonferroni correction or multiple rang comparison) was performed for comparisons between the different etiological groups. Inflammatory markers that were found to differ significantly between the groups (BP vs. VP, BP vs. AP, and VP vs. AP) were then further tested via receiver operating characteristic (ROC) curve analysis in order to estimate its usefulness in a differential diagnosis; the area under the curve (AUC) was calculated with 95% confidence intervals (95% CI), and optimal cut-off values were calculated with the use of the Youden index; the corresponding sensitivity, specificity, positive predictive value (PPV), and the negative predictive value (NPV) were shown. In order to facilitate the comparisons between the AUC if an inverse relationship was observed (i.e., destimulating effect instead of a stimulating one), it was highlighted and the re-calculated AUC (over 0.5) was presented. 

A *p*-value lower than 0.05 was acknowledged to be statistically significant. The statistical analysis was performed using Statistica 13.1 software (Statsoft, Tulsa, OK, USA). The study obtained approval from the Ethics Committee at the Centre of Postgraduate Medical Education in Warsaw (approval number 14/2021; issued on 10 March 2021).

## 3. Results

**Figure 1:**
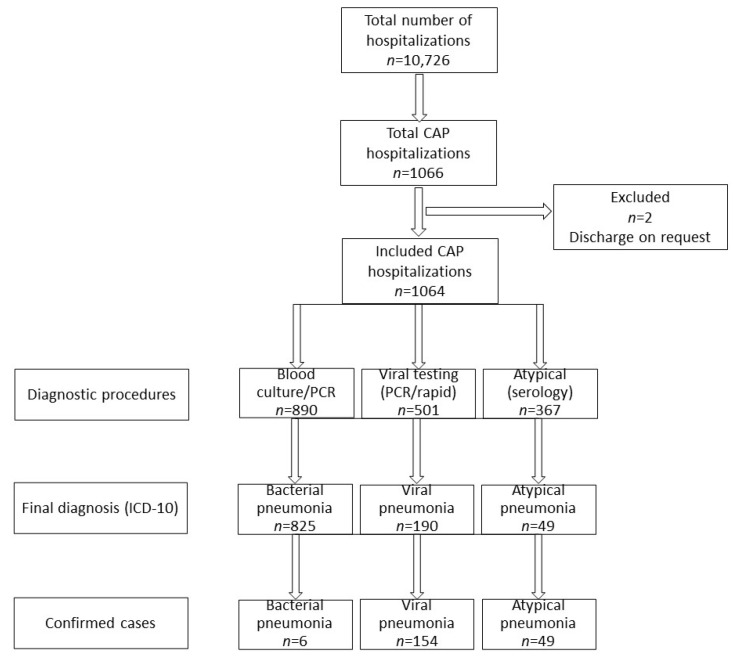
A flowchart of patients in the study. Note, some of the patients underwent diagnostic procedures towards more than one conditions, but finally each patient was diagnosed with onlly one condition. CAP—community-acquired pneumonia; ICD-10—International Classification of Diseases, Tenth Revision (ICD-10).

In the period between 2013 and 2018, a total number of 10,726 hospitalizations took place; finally, 1064 children (591 boys, 473 girls) with CAP were included in the study (Figure 1). The median age was 29.2 months (IQR: 13.9–54.8), with the following age distribution: 76 patients under 3 mo (7.2% of the total number of patients), 167 aged 3–11 mo (15.6%), 597 aged 12–59 mo (56.1%), and 224 aged 5 years and older (21.1%). The median LOS was 7 days (IQR: 5–9).

### 3.1. Etiology of Childhood Pneumonia

The bacterial/possibly bacterial pneumonia was diagnosed in 825 children (77.5%), while the viral/possibly viral one in 190 (17.9%), and the atypical one in 49 (4.6%) patients. 

**Figure 2:**
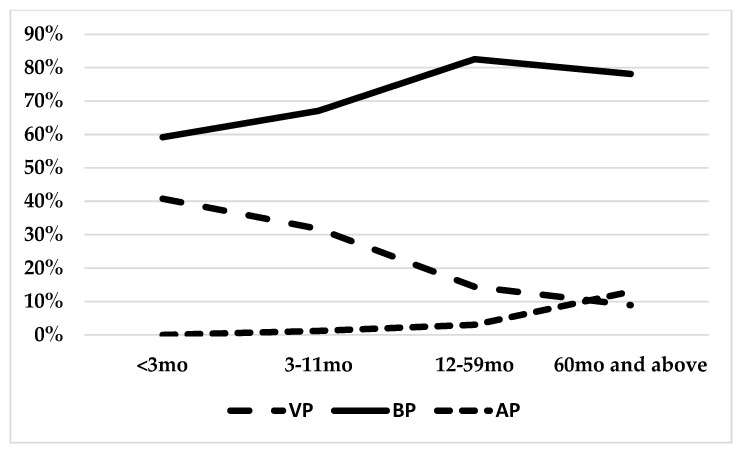
Frequency of pneumonia etiological factors in different age groups (VP—viral pneumonia; dashed line; BP—bacterial pneumonia, solid line; AP—atypical pneumonia, dotted line).

The proportion of the particular diagnoses varied among the groups; VP was recognized mainly in the youngest patients (under 3 mo) in 41% of cases, and decreased with the patients’ age: 32% in 3–11 mo, 14% in 12–59 mo, and 9% in ≥ 60 mo (Figure 2).

On the other hand, the percentage of AP was the highest in ≥60 mo (13%), and correlated with the age (3% in 12–59, 1% in 3–11 mo, and no cases in the youngest age group). Bacterial pneumonia (confirmed or suspected) was diagnosed in the vast majority of cases, with the highest contribution in 12–59 mo (83%), and slightly lower in ≥60 mo (78%). The patients’ age differed significantly among the groups, with the youngest children in the VP group (median 16.6, IQR: 4.8–35.5 months), followed by the BP (median 30.9, IQR: 16.1–55.3), and the oldest children in the AP group (median 75.2, IQR: 43.4–108,5). Patients with VP required a slightly longer LOS than BP patients (Table 2).

The etiological factor was established in 209 children (20%), and influenza was the leading cause (82 cases: 39% of the diagnosed cases and 7.7% of the total number of cases), followed by RSV (72 cases: 35% and 6.8% of cases, respectively), *Mycoplasma pmeumoniae* (19%, 39 cases), *Chlamydophila pneumoniae* (8 cases), *Streptococcus pneumoniae* (2 cases), *Bordetella pertussis* (2 cases), and *Streptococcus pyogenes* (1 case), *Streptococcus mitis/oralis* (1 case), *Streptococcus parasanguinis* (1 case), and *Staphylococcus warneri* (1 case). In total, 154 cases of viral, 49 atypical, and 6 bacterial infections were confirmed. A difference between the median age was seen only between viral and atypical pneumonia (16 vs. 75.2 months), and the differences in terms of the LOS were noted.

### 3.2. Inflammatory Markers

Children with BP presented the highest white blood cell count on admission (median 12.82 × 10^3^/μL; IQR: 9–18.4), but a significant difference was observed only between the BP and the VP (median 10.95 × 10^3^/μL; IQR: 7.3–15.1). The absolute neutrophil count differed between the BP (median 7.1 × 10^3^/μL; IQR: 3.9–12.1) and the VP (median 4.41 × 10^3^/μL; IQR: 2.3–8.1), and between the VP and the AP (median 6.4 × 10^3^/μL; IQR: 4.8–11.6), but not between the BP and the AP. Among the serum inflammatory markers, procalcitonin differed among all the groups; the highest values were observed in the BP (median 0.36 ng/mL; IQR: 0.12–1.5), followed by the VP (median 0.22 ng/mL; IQR: 0.1–0.52), and the AP (median 0.11 ng/mL; IQR: 0.08–0.16), whereas the CRP differed only between the bacterial and viral pneumonia (24.26 vs. 7 mg/L, with IQR: 7.7–66.9 and 2.3–22.7, respectively). A statistical significance in the CRP to PCT ratio was observed among all the groups, with the highest value in the AP group (median 120.4; IQR: 65.3–190.1), followed by the BP (55.9, IQR: 19.2–141.8), and VP (36.5, IQR: 13–83.9) (Table 2). 

In the ROC analysis, inflammatory markers that differed between the groups were further tested to investigate the potential of a differential diagnosis. 

The highest AUC for the distinction between bacterial and viral pneumonia was observed for CRP (AUC = 0.675; 95% CI: 0.634–0.715; *p* < 0.01), followed by the ANC (AUC = 0.658; 95% CI: 0.616–0.7; *p* < 0.01), WBC (AUC = 0.606; 95% CI: 0.562–0.649; *p* < 0.01), CRP/PCT (AUC = 0.592; 95% CI: 0.545–0.638; *p* < 0.01), and PCT (AUC = 0.589; 95% CI: 0.545–0.633; *p* < 0.01) (Table 3). 

To differentiate between the typical and atypical bacteria, the PCT showed the best performance (AUC = 0.73; 95% CI: 0.67–0.794; *p* < 0.01), followed by CRP/PCT (AUC = 0.66; 95% CI: 0.58–0.73; *p* < 0.01), and CRP (AUC = 0.604; 95% CI: 0.54–0.67; *p* < 0.01). 

With regard to a viral versus atypical etiology, the CRP/PCT (reverse relationship) showed the best performance (AUC = 0.752; 95% CI: 0.67–0.83; *p* < 0.01), followed by PCT (AUC = 0.68; 95% CI: 0.6–0.76; *p* < 0.01), the ANC (reverse relationship—AUC = 0.67; 95% CI: 0.59–0.75; *p* < 0.01), and CRP (AUC = 0.607; 95% CI: 0.53–0.69; *p* < 0.01).

In general, inflammatory markers showed promising positive predictive values at optimal cut-offs calculated in the analysis (88.8% for CRP in BP-VP, 98.3% for PCT in BP-AP, and 91.6% for CRP/PCT in VP-AP) at the cost of a low NPV (29.5%, 13.1%, and 40.7%, respectively).

In a separate analysis, focusing on the confirmed etiological factors only, the results were comparable to those from the whole study group with regard to the possible use of corresponding inflammatory markers, although generally, higher AUC values were noted since they may have overestimated the true usefulness of the markers in clinical settings (Table S1). 

The highest PCT values were observed for bacterial infection (median 8.4 ng/mL; IQR: 6.2–21.3), followed by viral infection (median 0.25 ng/mL; IQR: 0.1–0.71), and atypical infection (median 0.11 ng/mL; IQR: 0.08–0.16), while the CRP differed only between bacterial and viral pneumonia (median 264.3 mg/L versus 8.5 mg/L). The CRP to PCT ratio differed between atypical and viral pneumonia (120.4 vs. 38, respectively). The white blood cell count did not differ between the groups, while the ANC was higher in children with atypical pneumonia compared to those with viral pneumonia (6.4 vs. 4.7 × 10^3^/μL).

### 3.3. Pneumonia Impact on Hospitalizations

The total burden of pneumonia was significant, affecting both the number of hospitalizations and, to a greater extent, the number of patient days due to a longer LOS (7 days versus 4.5 days in controls). The number of CAP patients (***n*** = 1064) corresponded to 9.92% of the total number of hospitalizations (***n*** = 10,726), while the contribution to the total number of patient days was almost 1.5-fold higher, and reached 14.8% (7587 out of 51,381 patient days). According to age, the CAP in infants accounted for 6.8%, with a 10.6% share in patient days, whereas in children aged 1 and older, the share in the number of hospitalizations and patient days reached 11.4% and 17.5%, respectively.

## 4. Discussion

Our study included 10,726 hospitalizations with 1064 CAP cases, which accounted for approximately 10% of the total number of hospitalizations and 15% of patient days at the pediatric ward, and the discrepancy results mainly from a longer length of stay. Although the number of pneumonia cases globally decreased by 22% between 2000 and 2015, the number of hospitalizations increased 2.9-fold [6]; this phenomenon might be attributable to changing epidemiology, but also varying approaches towards pediatric pneumonia, including increased concerns both in parents/tutors, as well as health-care specialists. Thus, every effort must be made to judiciously and safely decrease the length of stay in order to decrease the patients’ exposure to the hospital environment and to increase bed availability. 

One of the most challenging issues in the field of pediatrics is etiology confirmation in patients with pneumonia; the problem with various diagnostic strategies and possibilities remains to be solved, since eagerness to find etiological factor, on the one hand, is balanced by a tendency towards the least invasive diagnostic methods in children, on the other hand. Moreover, while the lack of confirmation of any particular etiological agent does not exclude its presence, a high percentage of multiple microorganisms might also be observed in healthy children, and a differentiation between infection and colonization needs to be considered [41]. A strong emphasis needs to be placed on the age-related variations in the frequency of the particular etiological agents. A comprehension of these dependencies may ameliorate the use of antimicrobials and the clinical approach in everyday practice. Viral/possibly viral etiology was found in 17.9% of cases in our study; these results are in line with a Taiwanese study, in which viral pneumonia accounted for 17.4% of pneumonia cases [11], while other researchers have report varied data, ranging from 8.4% in a Korean study to 70.4% in data from Ecuador [12,14]. Nevertheless, some generalizations may be made. Viruses cause pneumonia mainly in the youngest groups of patients; in our series, it was the leading cause in children younger than 3 mo (41% of cases) with a median age of 16.6 months, and studies from Norway, the USA, and Taiwan underline that viral pneumonia is the most common in children aged 2 and less [11,16,42]. Moreover, the prevalence of viral pneumonia decreases with age; we found a decrease from 32% in 3–11 mo, to 14% (12–59 mo) and 9% (5 years and older), and a decreasing trend was also observed in studies from Egypt, the USA, Norway, and Taiwan [11,15,16,42]. Among viral infections, patients were diagnosed most commonly with influenza and RSV, which accounted for 7.7% and 6.8% of the total number of cases, respectively. In other reports, RSV frequency in children hospitalized due to pneumonia varied hugely, from 7.2% [11], to 19.4% [36], 38% [43] and 39.9% [44], and this mainly depends on the study setting, seasonality and local variations. Influenza, which is the major diagnosed single cause of pneumonia, is reported in 4.3% [36], 7.2% [11], and 10.7% of cases [44], which is generally in line with our data. Here, the proportion seems to reflect the true occurrence more accurately than in the case of RSV.

In the case of RSV, the huge discrepancies might be attributable to the clinical diagnosis of pneumonia; RSV is the most frequent cause of bronchiolitis, but chest X-ray reveals many minor abnormalities in patients diagnosed with RSV bronchiolitis, which are often diagnosed as pneumonia [45,46,47]. 

Typical bacteria, on the other hand, seem to show a fairly constant prevalence throughout various age groups; a huge study from the USA reports *S. pneumoniae* in approximately 3 to 4% of patients aged < 2 years old, 2–4, 5–9, and 10–17 years old [43].

Although the most commonly recognized, typical pneumonia has been confirmed in only a few cases, which is possibly related to the low sensitivity of the available diagnostic methods. Blood cultures reveal positive results in approximately 2.5–15% of samples from hospitalized patients, while our previous analysis showed positive blood cultures in 0.6% of cases [17,19,20,48]. Depending on the study location, group selection, and diagnostic methods used, results vary hugely. A preliminary study from Egypt and a study from China found bacteria to be the most common causes of pneumonia in children of all age groups [13,15], while other research finds viruses in the vast majority of cases (at least one virus in over 70% cases) [43]. It should also be remembered that the introduction of vaccines against *Haemophilus influenzae* type B and, even more importantly, against pneumococci, decreased the frequency of bacterial pneumonia, especially more severe cases, simultaneously increasing the proportionate contribution of viruses [49]. The presence of non-vaccine pneumococci serotypes and viral–bacterial coinfections contribute to the limited reduction in pneumonia, and as a consequence, a change in the clinical pattern of pneumonia may be expected due to a higher contribution of viral pneumonia [50], to viral + bacterial co-infection and to non-vaccine serotypes of pneumococcus. Nevertheless, special attention should be paid to the high number of cases diagnosed as potentially bacterial without confirmation, especially in the oldest age group of children. First, group selection needs to be emphasized—we analyzed the frequency of diagnoses only in hospitalized children, in whom a higher frequency of bacterial pneumonia might be expected due to its severity. While in the youngest groups of patients (i.e., up to 3–6 months old, depending on local guidelines), hospitalization is indicated in the case of pneumonia, irrespective of the disease severity, in older patients, it is based upon clinical indications, which, in general, reflect a more severe disease course [40]. Second, it might reflect the problem of antibiotic overuse, and different diagnostic tests (including rapid antigen tests) that prove the viral origin of infection help to decrease the use of antibiotics by reducing anxiety in terms of treatment side effects [51,52]. Bacteria may cause coinfection or suprainfection of s primarily viral infection, resulting in the need for antibiotic treatment, and older children might be more vulnerable to bacterial suprainfections [53]. Additionally, due to the limited diagnostic procedures, some cases of viral or atypical pneumoniae might have been missed. Nonetheless, we found atypical bacteria in up to 20% of confirmed cases and in 5% of the total number of cases, while other research suggests an atypical etiology in 10–30% of CAP, mainly caused by *M. pneumoniae* [21]. The percentage of atypical pneumonia correlated with age, and the highest proportion was observed in children over 5 years old (13%), which is in line with other studies indicating that this etiology usually occurs among children above 5 years of age; however, several recent studies also emphasize its prevalence in younger children [13,21,54]. The results, however, need to be appraised with caution, since we only used the serological confirmation of *M. pneumoniae* and *C. pneumoniae* infection, and, especially in the latter case, cross-reactivity, as well as impaired specificity, might be observed [55].

For the purposes of differentiation between viral and bacterial pneumonia, the CRP showed a higher AUC than the procalcitonin (AUC = 0.675 vs. AUC = 0.589, respectively). Data on using the CRP remain controversial. While some studies show a significant association between higher CRP and a bacterial etiology [56,57], others find no difference in CRP levels [58,59]. A meta-analysis by Flood et al. [60] proved only a weak ability of CRP to predict a bacterial etiology. The AUC for CRP found in our study is similar to the results obtained by Esposito et al. [61] in a study that enrolled 433 children with CAP (0.66, 95% CI 0.61–0.71), in which the AUC for PCT was slightly higher (0.69, 95% CI 0.63–0.75), while a much better diagnostic performance of procalcitonin was reported by Moulin et al. [62] (AUC = 0.93 and 95% CI 0.85–0.97 for PCT; AUC = 0.84 and 95% CI 0.73–0.91 for CRP). Nevertheless, it must be remembered that different study settings, inclusion criteria, end-points or even definitions of pneumonia may significantly influence the results. Future studies focusing on the usefulness of PCT are guaranteed, especially in terms of its negative predictive value [36]. We observed no differences regarding the leukocytes or neutrophils for BP–VP differentiation, which is in line with studies by Virkki and Korppi that disproved these markers for this particular use [56,63].

Our study also confirms the observation in other studies that PCT may help to differentiate a typical CAP from an atypical one (AUC = 0.73; 95% CI: 0.67–0.794; *p* < 0.01) [35,36,38,64,65]. The study of Stockmann showed much higher median PCT serum levels in children with typical bacteria (6.10 ng/mL; IQR, 0.84–22.79), compared to atypical pneumonia (0.10 ng/mL; IQR, 0.06–0.31) [36], similarly to studies on adults that report higher PCT in typical than atypical pneumonia [38,64,65]. In a study by Neeser et al. [38], the PCT differentiating potential (AUC of 0.85) was outweighted by the CRP/PCT ratio (AUC of 0.91), which in our research was AUC = 0.66.

The CRP/PCT ratio shows a promising value in distinguishing an atypical etiology from a viral one (AUC = 0.752; 95% CI: 0.67–0.83). These results align with the Swiss study on adult patients with CAP, where a much higher CRP/PCT ratio was seen in *Mycoplasma pneumoniae* [38] than in viral CAP (500 mg/μg, IQR 380–1000 vs. 188 mg/μg, IQR 86–385, *p* < 0.001). We observed a slightly worse performance (AUC = 0.68; 95% CI: 0.6–0.76) of PCT concentrations; lower PCT values in the course of atypical CAP were also reported by Stockmann [36] and by Wang in the case of children with hospital-acquired pneumonia [66], while in adults, Kruger found no significant differences in PCT concentrations [67].

There are strengths and limitations to this study. First, it needs to be recognized that the study analyzed a single pediatric ward only, and any generalizations should be made with extreme precaution. Second, the etiological factor was established in only 20% of cases, which might be attributed to a lack of specific test in some of the cases (not all the patients underwent the same diagnostic tests); thus, a different pathogen distribution across the age groups cannot be excluded, and this study might only provide hints regarding the etiology of the individual pneumonia cases. We did not use multiple pathogen panels, which would facilitate the detection of coinfections, especially viral ones, that need to be taken into consideration. Similarly, the usefulness of the inflammatory markers might differ in other study settings, given the obstacles met regarding the confirmation of the pneumonia etiology, and we aimed to verify a practical approach. It should be remembered that inflammatory markers in our study showed low to moderate test accuracy. 

## 5. Conclusions

In conclusion, the impact of CAP on hospital treatment is far beyond what the crude number of hospital admissions suggests. We observed that viral pneumonia decreases with age, in contrast to the percentage of atypical pneumonia, while bacterial pneumonia remained constant in all age groups. A major impact of two viruses (influenza and RSV) is clearly seen—they were confirmed in approximately 15% of the total number of CAP cases; on the other hand, the vast majority of cases were finally diagnosed as bacterial or possibly bacterial pneumonia, but only 0.6% found a laboratory confirmation with the use of the currently employed techniques. Diagnostic possibilities, although narrow, may be supported by procalcitonin levels (the highest increase in bacterial infections, followed by viral and atypical CAP). The CRP may also be used, yet its increase only differentiates bacterial from viral pneumonia, while the CRP/PCT ratio points to patients with an increased risk of atypical infection. Efforts to establish the etiology of CAP are crucial in order to implement adequate treatment.

## Figures and Tables

**Table 1:** Inclusion and exclusion criteria.

Inclusion Criteria	Exclusion Criteria
1. Age: 0–18 years old 2. Hospitalization due to CAP between January 2013 and December 2018 3. CAP criteria: (a) community-acquired pneumonia: signs/symptoms (see below) present prior to hospitalization or up to the first 48 h after hospital admission (b) 2 or more of the following signs/symptoms: fever (38 degrees Celsius or more), cough, tachypnea (according to age, i.e., 0–1 months > 60 breaths/min, >1–12 months > 50 breaths/min, >12–59 months > 40 breaths/min, 60 months or older > 25 breaths/min), intercostal retractions, a dull percussion note and (c) crackles or a bronchial murmur on auscultation OR positive chest X-ray (i.e., presence of consolidation, or parenchymal infiltrates, or linear densities, or patchy densities, or pleural effusion) OR positive lung ultrasound (hypoechogenic lung lesions, or pleural line abnormalities—locally absent or hypoechogenic, or bronchogram sign-hyperechogenic area within the consolidation, or impaired lung respiratory mobility-absent or decreased “lung sliding”)	1. immunodeficiency (congenital or acquired or drug-related), 2. hemodynamically significant heart disease 3. disease worsening the course of respiratory tract infection (cystic fibrosis, neuromuscular disease) 4. lack of full knowledge on the clinical course of the disease (e.g., a discharge on parent’s/tutor’s request)

**Table 2:** Baseline characteristics of the study group. Non-parametric Kruskal–Wallis with corresponding post hoc test (multiple range comparison) was used to verify statistically significant differences (shown in the right column).

	VP (*n* = 190)			BP (*n* = 825)			AP (*n* = 49)			
	Median	IQR		Median	IQR		Median	IQR		Statistical Significance
age (months)	16.63	4.77	35.47	30.90	16.13	55.30	75.15	43.43	108.46	VP vs. BP vs. AP
WBC (10 × 3/μL) ***n*** = 1061	10.95	7.30	15.13	12.82	8.99	18.40	12.27	8.49	15.50	VP vs. BP
ANC (10 × 3/μL) ***n*** = 1061	4.41	2.33	8.08	7.10	3.91	12.14	6.39	4.82	11.59	VP vs. BP VP vs. AP
CRP (mg/L) ***n*** = 1061	7.07	2.33	22.66	24.26	7.67	66.94	14.57	6.32	32.34	VP vs. BP
PCT (ng/mL) ***n*** = 936	0.22	0.10	0.52	0.36	0.12	1.50	0.11	0.08	0.16	VP vs. BP vs. AP
CRP/ PCT ***n*** = 936	36.46	12.98	83.90	55.89	19.19	141.78	120.41	65.33	190.06	VP vs. BP vs. AP
LOS (days)	7	5	10	7	4	9	7	5	10	VP vs. BP

BP—bacterial pneumonia; VP—viral pneumonia; AP—atypical pneumonia; IQR—interquartile range; WBC—white blood cells; ANC—absolute neutrophil count; CRP—C-reactive protein; PCT—procalcitonin; CRP/PCT—CRP to procalcitonin ratio; LOS—length of stay.

**Table 3:** Inflammatory markers in differentiation of pneumonia etiology—the results of the ROC-curve analysis. BP—bacterial pneumonia; VP—viral pneumonia; AP—atypical pneumonia; AUC—area under the curve; 95% CI—95% confidence interval; PPV—positive predictive value; NPV—negative predictive value. Inflammatory markers: WBCs—white blood cells; ANC—absolute neutrophil count; CRP—C-reactive protein; PCT—procalcitonin; CRP/PCT—CRP to procalcitonin ratio. Statistically non-significant values written in italics.

BP vs. VP									
	AUC	95% CI		*p*	Optimal Cut-Off (Youden Index)	Sensitivity (95% CI)	Specificity (95% CI)	PPV (95% CI)	NPV (95% CI)
WBC	0.606	0.56	0.65	<0.01	11.96	56.33%	59.47%	85.74%	23.94%
						52.86% to 59.75%	(52.13% to 66.52%)	(83.36% to 87.83%)	(21.47% to 26.60%)
ANC	0.658	0.62	0.70	<0.01	5.22	65.69%	58.42%	(87.24%	28.24%
						62.34% to 68.94%	(51.06% to 65.51%)	85.15% to 89.07%)	(25.25% to 31.44%)
CRP	0.675	0.63	0.72	<0.01	12.94	64.56%	64.55%	88.78%	29.54%
						61.17% to 67.83%	(57.28% to 71.36%)	(86.64% to 90.61%)	(26.71% to 32.54%)
PCT	0.589	0.55	0.63	<0.01	0.33	52.12%	66.46%	87.59%	23.41%
						48.43% to 55.80%	(58.60% to 73.70%)	(84.88% to 89.86%)	(21.11% to 25.89%)
CRP/PCT	0.592	0.55	0.64	<0.01	74.332	43.82%	73.12%	88.12%	22.24%
						40.18% to 47.51%	(65.55% to 79.82%)	(85.01% to 90.66%)	(20.34% to 24.27%)
BP vs. AP									
	AUC	95% CI		*p*	Optimal Cut-Off (Youden Index)	Sensitivity (95% CI)	Specificity (95% CI)	PPV (95% CI)	NPV (95% CI)
WBC	*0.563*	*0.49*	*0.64*	*0.1047*					
ANC	*0.507*	*0.43*	*0.58*	*0.8582*					
CRP	0.604	0.54	0.67	<0.01	22.19	52.50%	69.39%	96.64%	8.02%
						49.02% to 55.96%	(54.58% to 81.75%)	(94.94% to 97.78%)	(6.67% to 9.62%)
PCT	0.733	0.67	0.79	<0.01	0.187	64.16%	82.22%	98.32%	12.37%
						60.56% to 67.64%	(67.95% to 92.00%)	(96.89% to 99.10%)	(10.68% to 14.30%)
CRP/PCT	0.656	0.58	0.73	<0.01	65	53.16%	77.78%	97.48%	9.31%
						49.46% to 56.83%	(62.91% to 88.80%)	(95.71% to 98.53%)	(7.94% to 10.89%)
VP vs. AP									
	AUC	95% CI		*p*	Optimal Cut-Off (Youden Index)	Sensitivity (95% CI)	Specificity (95% CI)	PPV (95% CI)	NPV (95% CI)
WBC	*0.449*	*0.36*	*0.54*	*0.2434*					
ANC	0.667	0.59	0.75	<0.01	4.42	50.53%	79.59%	90.57%	29.32%
						43.19% to 57.84%	(65.66% to 89.76%)	(84.44% to 94.44%)	(25.32% to 33.67%)
CRP	0.607	0.53	0.69	<0.01	69.17	9.52%	95.92%	90.00%	21.56%
						5.74% to 14.63%	(86.02% to 99.50%)	(68.36% to 97.40%)	(20.33% to 22.84%)
PCT	0.68	0.60	0.76	<0.01	0.19	22.98%	95.56%	94.87%	25.75%
						16.73% to 30.26%	(84.85% to 99.46%)	(82.26% to 98.66%)	(23.79% to 27.81%)
CRP/PCT	0.752	0.67	0.83	<0.01	63.895	68.12%	77.78%	91.60%	40.70%
						60.30% to 75.26%	(62.91% to 88.80%)	(86.20% to 95.01%)	(34.26% to 47.47%)

## Data Availability

Data are available on request from the authors.

## Supplementary Materials

The following supporting information can be downloaded at: https://www.mdpi.com/article/10.3390/jcm11195506/s1, Table S1: The results of the ROC curve analysis on the usefulness of the inflammatory markers with regard to the differentiation between bacterial pneumonia (BP), viral pneumonia (VP), and atypical pneumonia (AP).
