# Supplementary material for: The Etiology of Community-Acquired Pneumonia Correlates with Serum Inflammatory Markers in Children
Source: J Clin Med. 2022 Sep 20;11(19):5506. doi: 10.3390/jcm11195506 (PMC9571658; doi:10.3390/jcm11195506)
Supplement: Supplementary file 1 [file jcm-11-05506-s001.zip › jcm-1872698-supplementary.pdf]

Table S1. The results of the ROC curve analysis on the usefulness of the inflammatory markers with regards to the differentiation between bacterial pneumonia (BP), viral pneumonia (VP), and atypical pneumonia (AP). AUCs are presented with 95% confidence intervals. *Optimal cut-off points were calculated with the Youden index. Abbreviations: AUC- area under the curve, 95%CI- 95% confidence interval, PPV- positive predictive value, NPV- negative predictive value, WBC- white blood cells, ANC- absolute neutrophil count, CRP- C-reactive protein, PCT- procalcitonin, CRP/PCT- CRP to procalcitonin ratio. ns- statistically non-significant

BP vs VP									
	AUC	95% Lower limit	95% Upper limit	p	optimal cut-off value*	Sensitivity (95%CI)	Specificity (95%CI)	PPV (95%CI)	NPV (95%CI)
WBC				ns					
ANC				ns					
CRP	0.806	0.516	1.000	0.0389	237.86	66.67%	100.00%	100.00%	98.72%
						22.28% - 95.67%	97.63% - 100.00%		96.13% - 99.58%
PCT	0.939	0.844	1.000	0.0000	6.21	80.00%	97.81%	57.14%	99.26%
						28.36% - 99.49%	93.73% - 99.55%	28.61% - 81.60%	95.87% - 99.87%
CRP/PCT				ns					
BP vs AP									
WBC				ns					
ANC				ns					
CRP	0.810	0.516	1.000	0.0387	47.46	83.33%	85.71%	41.67%	97.67%
						35.88% - 99.58%	72.76% - 94.06%	24.79% - 60.76%	87.49% - 99.61%
PCT	0.969	0.921	1.000	0.0000	0.59	100.00%	93.33%	62.50%	100.00%
						47.82% - 100.00%	81.73% - 98.60%	35.84% - 83.26%	
CRP/PCT	0.849	0.725	0.973	0.0000	80.17	100.00%	66.67%	25.00%	100.00%
						47.82% - 100.00%	51.05% - 80.00%	18.07% - 33.51%	

AP vs VP									
WBC				ns					
ANC	0.664	0.582	0.746	0.0001	3.73	87.76%	40.91%	32.09%	91.30%
						75.23% - 95.37%	33.06% - 49.11%	28.54% - 35.85%	82.89% - 95.79%
CRP				ns					
PCT	0.692	0.607	0.776	0.0000	0.18	80.00%	59.85%	39.56%	90.11%
						65.40% - 90.42%	51.14% - 68.13%	33.74% - 45.70%	83.33% - 94.32%
CRP/PCT	0.752	0.670	0.833	0.0000	65.1	77.78%	66.42%	43.21%	90.10%
						62.91% - 88.80%	57.86% - 74.26%	36.45% - 50.23%	83.87% - 94.09%
